# Rapid stream stimulation can enhance the stimulus selectivity of early evoked responses to written characters but not faces

**DOI:** 10.1371/journal.pone.0213637

**Published:** 2019-03-15

**Authors:** Canhuang Luo, Wei Chen, Ye Zhang, Carl Michael Gaspar

**Affiliations:** 1 Institutes of Psychological Sciences, Hangzhou Normal University, Hangzhou, China; 2 Zhejiang Key Laboratory for Research in Assessment of Cognitive Impairments, Hangzhou, China; 3 Center for Cognition and Brain Disorders, Hangzhou Normal University, Hangzhou, China; 4 Objects and Knowledge Laboratory, New York University Abu Dhabi, Abu Dhabi, UAE; French National Center for Scientific Research (CNRS) & University of Lyon, FRANCE

## Abstract

The recognition potential (RP) is an early visually evoked response (~250 ms) whose magnitude is sensitive to object recognizability and related factors. The RP is often measured when objects are embedded in a rapid stream of masking stimuli (the RSS paradigm), especially in reading research. The idea is that RSS provides greater stimulus-dependent variations in RP, compared to the corresponding variations without RSS. However, this idea has never been subject to systematic evaluation. We directly test whether RSS can enhance 2 types of RP stimulus selectivity, by measuring the RP in conditions that only differ in the presence or absence of a masking stream and in the type of stimulus shown. We measure the effect of image inversion on RP for Chinese characters (experiment 1); the effect of orthographical correctness on RP for Chinese characters (experiment 3); and as a control study, the effect of image inversion on the N170 response to faces (experiment 2). To ensure a fair comparison, the earliest negative deflections (RP/N170) measured with and without RSS should at least have similar channel ranges, and topographical distributions of both amplitude and selectivity. Our first set of results clearly supports this. Our main results clearly show an increase in stimulus-selectivity for RSS over non-RSS in both of the RP (Chinese character) experiments, but no such enhancement in the N170 (face) experiment. This provides incentive for investigations into the underlying mechanisms of selectivity enhancement. Our findings may also help to explain contradictory findings between RP/N170 studies that differ only in the use of noise masks, which is sometimes treated as trivial detail in papers that do not reference the RP/RSS literature.

## Introduction

The rapid-stream-stimulation procedure (RSS) is a paradigm for stimulus presentation that is thought to enhance early electrophysiological responses to recognizable stimuli. In this study, we test whether RSS increases the recognition potential (mostly measured in response to linguistic material), and whether it also affects selectivity measured with other stimuli (the N170 response to faces in this study).

The recognition potential (RP) is an electrical brain response peaking around 200 to 250ms, obtained when subjects view recognizable images, such as words [1–3] or pictures [4–5]. Variation in RP amplitude across categories of stimuli is a critical result, because it is taken to be an early index of neural selectivity. As RP has been adopted almost exclusively by reading researchers, most stimulus contrasts that have been studied involve words and word-like strings with varying types of linguistic content: words versus pseudo-words [6–8]; words versus non-words [6–9]; pseudo words versus non-words [1, 7]; animal versus non-animal words [6, 10]; congruent versus incongruent [11]; concrete versus abstract [7]; open-class versus closed-class [6]; and upright versus upside-down Chinese characters [12–13].

The N170 is an ERP component with a negative deflection that is evoked most strongly by familiar objects such as faces, words and cars (e.g., [14]). As described by its name, the N170 peaks around 170 ms after stimulus onset, and its amplitude is maximal at electrodes in the occipito-temporal area [15–16]. Since the N170 is larger for faces than for other categories, it is sometimes considered to be a face-specific component [15,17]. The N170 shares some important features with RP. Both are early components that are sensitive to object familiarity. Furthermore, both N170 and RP are observed at similar channels and have similar topography, i.e. the maximal peak can be observed in the occipital-temporal area of the scalp based on the topographies [7–8, 14, 16, 18–20]. Possible differences in the exact neural generators are described in the Discussion.

In almost all of the RP studies we have cited, the rapid-stream-stimulation procedure (RSS) is used to present stimuli, because it is thought enhance the degree of RP selectivity between perceptually and semantically different stimulus categories that vary in recognizability. However, there is no direct evidence that RSS enhances RP selectivity in the types of designs that are used in the vast majority of studies. This paper describes 2 experiments designed to provide a direct test of RSS-enhancement of RP selectivity to linguistic stimuli, and 1 additional study that examines the effect of RSS on the selectivity of the N170 response to faces.

The RSS procedure originates with Rudell [5], who devised this method as an efficient way to obtain very strong recognition-related ERP components, relative to the standard ERP paradigm. However, that original paper was based purely on theoretical considerations: he reports a number of RSS results, but no results obtained without RSS to serve as a control. Some reading researchers adopted the RSS procedure along with the assumption that RSS does provide better results than a standard ERP paradigm [3,21–24]. But as far as we know, no one has yet properly tested this assumption using a systematic experimental control.

Rudell himself devised the RSS based partly on an older paper from 1991 [2] that suggests a forward mask (or ‘preempt stimulus’) can increase the amplitude of the difference wave between recognizable and non-recognizable images (his original ‘recognition potential’). However, differences in paradigm (flanking noise versus streaming noise), procedure (e.g., electrode selection) and definition of RP (difference wave versus amplitudes of peaks) prevent us from extrapolating from that original result to the RP as it is studied today. A single subject was used for this experiment, and an attempt at replication has never been bothered with in the RP/RSS literature. Finally, Rudell [2] himself expresses surprise at the beneficial effect of masking. All of this suggests to us that further scrutiny of RSS-enhancement is not just important but also necessary.

Subsequent researchers have come up with additional reasons why RSS should provide a cleaner signal of RP [21–22], but these ideas, like the original theory of Rudell, remain untested. And strikingly, those hypotheses are motivated only by reference to either [5] or [2], whose shortcomings have already been noted. The closest evidence to date comes from a comparison of the RP lexicality effect between masking and non-masking conditions that was done ancillary to the main purpose of that paper, which was to provide a localization of the RP source. Dien et al. [9] find that the RP difference between words and non-words, measured using flanking masks, is reliably larger than the same difference of RP, measured without flanking masks. However, this mask-enhancement was not the main point of the paper. Dien et al., did not conduct a systematic within-subject comparison of RP topographies between masking and non-masking conditions, as we do in this paper. Finally, Dien et al. did not use the streaming of RSS which is the main masking paradigm that is believed to enhance RP [5, 21–22].

Aside from [9], there is only one additional paper that does suggest RSS may enhance RP [22]. However, the experimental design used in Iglesias et al. [22] makes it difficult to compare RSS and non-RSS conditions because they differ in both inter-stimulus timing and the presence of a masking stream. In the current study, we were careful to ensure that our RSS and non-RSS conditions differed only in the presence of a masking stream. Therefore, any enhancement of RP selectivity in our study can be solely attributed to RSS. Furthermore, Iglesias et al. [22] uses stimuli that are presented in the context of sentences; i.e., a sequence of centrally presented words that form a sentence. It is difficult to say whether RSS-enhancement of RP can generalize from those conditions to the isolated-target conditions that are more typical of RP studies. This is especially true as the explanation for enhancement provided in [22] differs from that provided elsewhere [2, 5, 21]. We acknowledge the valuable contributions of [9] and [22] but seek critical confirmation that RSS does enhance RP in the sort of stimulus conditions that are more typical of the literature. This may provide future researchers with a fuller range of options when considering RSS-enhancement and may also help as a starting point for empirical investigations into the exact causes of RSS-enhancement, which may be multiple.

Therefore, we compare RSS and non-RSS RP’s in 2 different types of object-processing experiment most appropriate for eliciting a recognizability response in Chinese subjects (the available subject pool at our institute in China). And unlike Dien et al. [9], we use the original version of RSS, where targets are temporally embedded in a stream of masks. This is important since RSS is by far the most common method used to elicit strong RP signals, as opposed to simple forward and backward masking [2, 9].

At this point, we’d like to emphasize that the goal of this study is to determine if RSS-enhancement of RP or N170 exists, and not to establish why it might exist. Nonetheless, possible mechanisms for RSS-enhancement should be briefly mentioned here to underscore the theoretical plausibility of RSS-enhancement. The most commonly provided explanation for why RSS might enhance RP requires a distinction between RP and earlier, purely visually-evoked components that might interfere with RP measurement. As Martín-Loeches [21] wrote: “The presence of background stimuli was originally introduced in order to avoid or reduce visual-related components such as the N1-P2 complex, as their latency largely overlaps with the RP. The background stimuli would act to preempt stimuli by temporally usurping activity in the visual afferent pathway.” While there is no formal model associated with this “pre-empt” explanation, the general idea of overlapping waveforms does seem like one that could potentially be tested. We propose one additional mechanism for RSS-enhancement, which simplifies the problem by ignoring the dynamical aspect of RSS and focusses solely on the effect of masking on stimulus information. It is possible that the forward masking introduced by RSS results in a trade-off between stimulus visibility and cognitive load. Note that both visibility and cognitive load may be positively related to strength of neuronal response. However, increased masking can have counteracting effects—reduced visibility (low neural response) but higher cognitive load (high neural response). Therefore, masking might in some cases increase neural responses if cognitive load is strengthened more than visibility is reduced. The relative merits of these two explanations are given in the Discussion. However, this paper’s primary concern is to provide a rigorous test of whether RSS-enhancement occurs in the first place.

Our experiments were conducted in China using Chinese subjects. To ensure recognizability, our stimuli were either Chinese characters (experiments 1 and 3), or photographs of real Chinese faces (experiment 2). To date, the only RP experiments using Chinese characters and subjects derive from two studies [12–13]. Experiment 1 in the current study examines one of the most robust stimulus contrasts employed between these two studies—upright versus upside-down (‘inverted’) characters [12]. This stimulus contrast is appealing for two reasons. First, it provides a conservative test of RSS-enhancement because the character orientation effect is already robust to begin with. Second, a contrast of stimuli that vary only in orientation ensures that the resulting variation in RP amplitude cannot be attributed low-level properties of the images. Instead, we can use RP selectivity as an index of sensitivity to higher level factors like ‘word-form analysis’ and semantic processing, both of which have been implicated in RP responsivity [1, 6–8, 10, 23].

As an additional study, experiment 2 contrasts N170 responses to upright and inverted Chinese faces. The focus on orientation selectivity of N170 in this experiment is motivated by the same reason provided for the first experiment—to rule out low-level image properties as an explanation for N170 sensitivity to face orientation. Compared to upright faces, the N170 is found to be enhanced for inverted faces indicated by a longer latency and larger amplitude [25–26].Our choice of face stimuli is to provide results that can help to understand the limits of RSS enhancement. The vast majority of RP studies use lexical stimuli like words, and various types of word-like strings. More specifically, all of the results that are consistent with RSS enhancement of RP selectivity have used lexical stimuli. Finally, faces are more commonly associated with the N170 response rather than the RP response and it is not clear whether similar mechanisms underlie RP and N170. Therefore, we feel more confident in describing the earliest negative deflection of the ERP response to faces as an N170, rather than an RP. Perhaps most importantly, our face stimuli are entirely unfamiliar to the participants and therefore lacking in the more specific exemplar recognizability of familiar Chinese characters and radicals. Taken together, we have weaker expectations that RSS will enhance N170 selectivity for face orientation, relative to our other two experiments (whose components are more conventionally labelled as RP).

Experiment 3 is perhaps the most strongly motivated by the previous literature and has the greatest expectation for RSS enhancement of RP selectivity. Chinese characters are of unique interest in detailing the specific forms of linguistic processing that may underlie early evoked responses. The reason for this is the mostly arbitrary mapping between visual and phonological forms. This property of characters therefore allows for the investigation of visual form familiarity with minimal involvement of phonology. To that end, Lin et al. [27] measured N170 responses to 4 types of Chinese character: real characters, pseudo characters, false characters and random strokes derived from real characters. In the left hemisphere, the only significant difference in N170 amplitude was between pseudo and false characters. This was the comparison of most interest to both Lin et al.[27] and to the current study (experiment 3). Pseudo characters are the combination of two radicals that never go together in real characters. False characters are similar but additionally, the two radicals are placed in relative positions that they never take in real characters (e.g., left radical to right position). While both pseudo and false characters share a similar ambiguity of meaning and phonology, false characters are less familiar in terms of their visual form. Thus, Lin et al. describe the lower N170 amplitude to false compared to pseudo characters as an effect of orthography. Interestingly, Chung et al.[28] compare these same two conditions and find no difference in N170 amplitude. As it turns out, Lin et al. [27] use a forward mask in their presentation sequence, but Chung et al. [28] do not use masking. Given that forward masking is claimed to be a critical aspect of the RSS-enhancement effect, we suspect that the forward mask used by Lin et al. [27] may have been critical for obtaining a robust orthography effect, similar to what is found in the RP literature (for example, see [8]). In fact, the types of stimulus contrasts employed by Lin et al. [27] are more prevalent in the RP literature than they are in the N170 literature. We suspect that this may have to do with the signal-enhancing effect of the forward mask that is assumed to occur in RP studies. We test that hypothesis here.

For those unfamiliar with RP research, it is important to point out that an early evoked response to a recognizable linguistic stimulus (first negative deflection) is almost always labelled an RP regardless of whether RSS has been employed or not; and regardless of whether the same component might be called an N170 by other researchers who may be unaware of the RP literature (e.g., [27–28]). This may be a bit confusing but, as far as our study is concerned, the exact label we attach to the first negative deflection of ERP is not critical for evaluating the worth of the current study’s results. Our main goal is to determine if RSS can enhance stimulus selectivity for the first negative deflection in the ERP. Experiments 1 and 3 use stimuli commonly associated with the RP literature (words and familiar parts of words with meaning), while experiment 2 uses stimuli more commonly associated with the N170 literature (faces). Expectation for RSS enhancement of early ERP selectivity might depend on the mechanism that underlies RSS enhancement. However, various forms of that mechanism have been proposed, some of which may apply equally well to both N170 and RP components.

**Fig 1:**
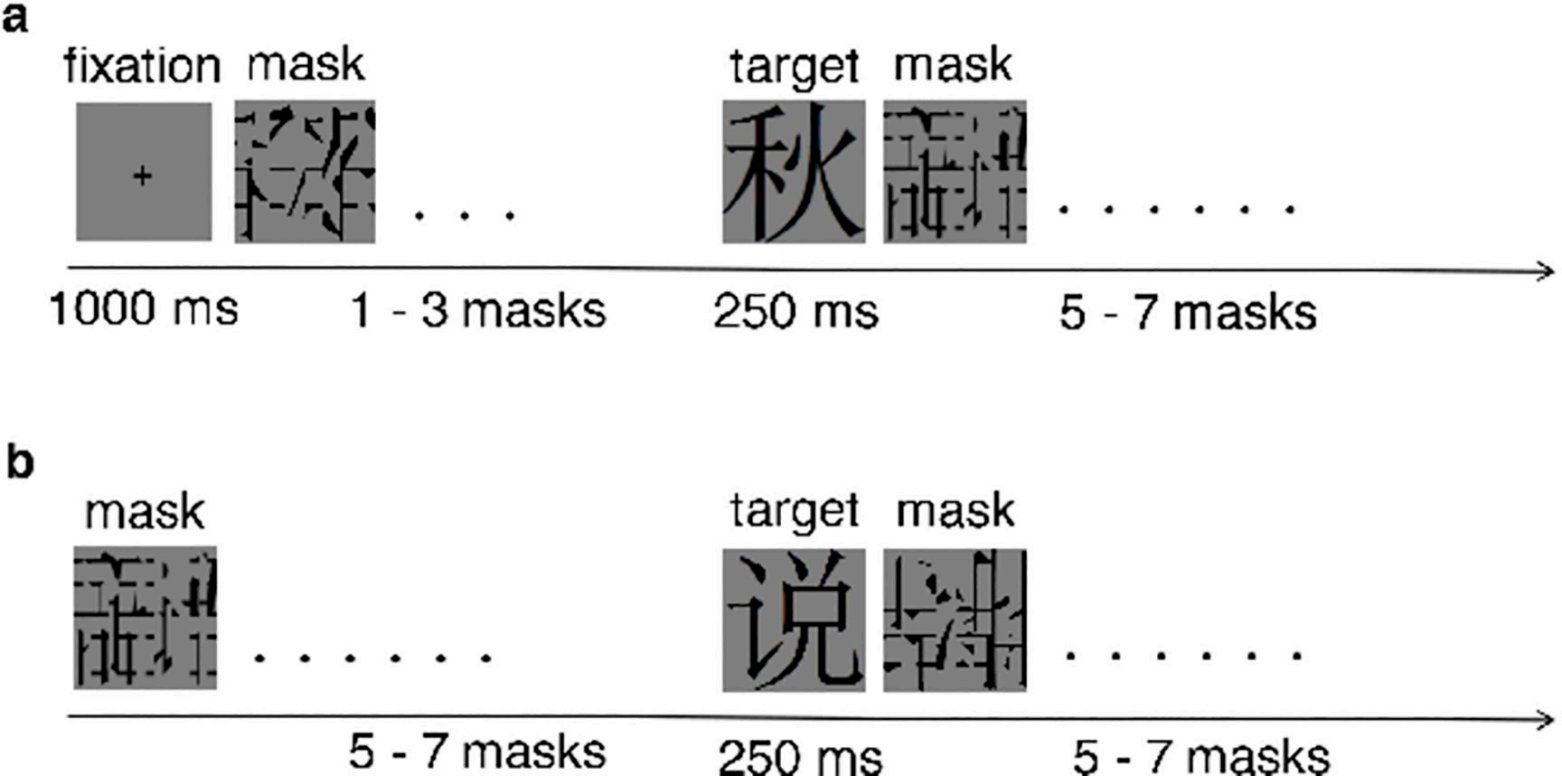
Stimulus sequences during the RSS experiment. The non-RSS is the same except that each masking stimulus is replaced with a blank screen, of equal duration. (**a**) The very first sequence of every block; (**b**) Every other sequence in a block, cycled with different target stimuli. All events last 250 ms, except for the fixation cross.

For each of the 3 experiments, subjects performed both an RSS (masked) and non-RSS (unmasked) version of that experiment on separate occasions. The procedure used for our RSS and non-RSS paradigms were as closely matched as possible. In fact, the only difference in procedure is that each presentation of a masking stimulus used in the RSS paradigm is replaced with a blank screen in the N170 paradigm, of equal duration (see Fig 1B). Therefore, any difference in result between paradigms can be wholly attributed to the presence or absence of a rapid sequence of masking stimuli, the critical feature of RSS [2,9]. These features of our experimental method are critical for ensuring a fair comparison between RSS and non-RSS results, and provide an important advance over the one study that comes closest to evaluating RSS-enhancement of RP [22]. The main independent variable we examine is the difference in RP/N170 component amplitude between types of stimuli. If RSS does enhance the stimulus-dependence of component amplitudes, then we expect a corresponding interaction between paradigm and stimulus type.

Given previous results, we can be more specific about the direction of some of our expected effects. In experiment 1, we expect upright characters to evoke an RP of higher amplitude than that evoked by inverted characters [12–13]. Critically, we expect this difference in RP amplitude to be greater in the RSS compared to the non-RSS conditions. However, this magnified difference could result from either an enhancement of the RP to upright characters, a dampening of the RP to inverted characters, or to both. Theories about the mechanism underlying RSS-enhancement are too variable to be any more specific [2,5,21–22]. In experiment 3, we expect the RP/N170 response to both real and pseudo characters to be of higher amplitude than the response to false characters [27]. However, it is possible that this amplitude difference is only found in the RSS condition. In this case, strong dependence of stimulus-selectivity on RSS could explain the apparently contradictory results of the two visual-word-form studies that were mentioned earlier [27–28]. For experiment 2, our expectations for results in the non-RSS condition are clear: the N170 should have a higher amplitude in response to inverted compared to upright faces [25–26]. However, our expectations for the results in the RSS condition are not as clear. While theories of RSS-enhancement of early components are somewhat variable, all of them predict a decrease in the amplitude difference between familiar and unfamiliar stimuli [2,5,21–22]. Whether responses to the familiar stimuli (faces) increase or responses to unfamiliar stimuli (inverted faces) decrease, the difference in amplitude should reduce because the initial conditions (non-RSS) are quite different from experiments 1 and 3: a higher amplitude response to the unfamiliar (inverted) stimuli. On the other hand, some researchers emphasize the role of RSS in enhancing the responses to linguistic stimuli in particular. If the main effect of RSS is to enhance the semantic signal associated with mainly linguistic material then we might expect no difference between RSS and non-RSS conditions in experiment 2.

As mentioned before, the RP measured in RSS is likely to occur in a later time window, due to the effects of the forward mask. If so, then this raises the possibility that the presence of noise, or some other aspect of the RSS procedure, has shifted the RP response to another electrode, another time-window, or both. Therefore, it is important that we establish that both types of RP—with and without RSS—share important aspects of topography and selectivity. It is also important that, in our evaluation of enhanced stimulus selectivity, we examine a range of likely electrodes. Therefore, the first half of our ERP results is a comparison of RP/N170 topography and timing between RSS and non-RSS paradigms.

## Methods

### Participants

All participants (13 females, 11 males, age range from 19 to 27 years with a mean of 21) in all experiments were Chinese students who participated as paid volunteers. They were all right-handed, had normal or corrected-to-normal vision and had no neurological history. The experiments reported here, the informed consent, and all experimental protocols, was approved by the Institutional Review Board of Hangzhou Normal University. All methods were performed in accordance with the approved guidelines and regulations. Informed consent was obtained from all subjects. Each participant signed a written, informed consent form prior to the ERP experiment and was compensated for CNY50 per hour after the experiment.

### Stimuli

#### Experiment 1

We selected 100 Chinese characters from the same database used in experiment 3 (the lexicality experiment). The frequency of these characters ranged from 877.7/million to 2536/million (Mean = 1462.3, SD = 462.1). The stroke number of the characters ranged from 7 to 11 (Mean = 8.6, SD = 1.3). The inverted characters were created by rotating the upright characters 180 degrees. The characters were grayscale (black on grey) and the visual angle was 4.4°x4.4°. The masks were created using the same method used in the lexicality experiment.

#### Experiment 2

Faces of neutral expression were taken from the CAS-PEAL-R1 face database [29]. We selected 100 faces (50 males) as stimuli. All faces were uniformly formatted within an oval with hair and moles removed with the customized MATLAB script and Photoshop. Then the luminance of faces is match across each face. The inverted faces were created by rotating the upright faces in 180°. The faces were grayscale and the visual angle was 4.5 by 6.6 degrees. The masks were created with the face stimuli by using the scramble filter on Photoshop, on which each face was cut into 20*20 pixel square then reconstructed randomly.

#### Experiment 3

We used real Chinese characters, pseudo characters and false characters as our stimuli, 100 characters per category. The real characters were selected from the Modern Chinese Corpus Centre for Chinese Linguistics, Peking University (http://ccl.pku.edu.cn:8080/ccl_corpus/index.jsp?dir=xiandai) and they were all left-right compound characters. Each pseudo character was created by combining the left radical of one real character, with the right radical of another real character; thus creating a novel character whose radicals are each in their correct position (i.e., orthographically correct). We looked through a Chinese dictionary (中华大字典) [30] to ensure that all pseudo characters were truly novel. False characters were made by first swapping the positions of each radical (incorrect orthography) of a real character, and then performing the cross-character re-combination done to produce pseudo-characters from real characters. Pseudo characters and false characters were made from the same real characters. Therefore, there were no differences of total stroke number and radical frequency among our three categories of stimuli. The frequency of real characters ranged from 117.4/million to 892.8/million (Mean = 421.8/million, SD = 191.3/million), and the stroke numbers of the three types of stimuli ranged from 7 to 11 (Mean = 8.9/million, SD = 1.3/million). Masking stimuli were created from the (real, pseudo and false) character stimuli by cutting each character into 16 equally-wide vertical strips and then randomizing their horizontal position. All characters are Song font, black and were presented on a grey background. The visual angle of stimuli are 4.4x4.4 degrees.

### Procedure

Subjects performed an RSS and non-RSS version of the same experiment in separate sessions, with order of session counterbalanced. Each session is further subdivided into blocks, with a brief rest period in between blocks. The exact stimuli and task varied across experiments, but the sequence of experimental events was exactly the same. During the RSS session, the very first sequence is a fixation sequence, as illustrated in Fig 1A: A fixation cross, 1 to 3 mask stimuli, a target, and 5 to 7 mask stimuli. This is followed by the main sequence (Fig 1B), which cycles until that block of epochs is complete: 5 to 7 mask stimuli, a target, then 5 to 7 mask stimuli. Each stimulus lasts for 250 ms, except for the fixation cross, which lasts 1 second. The average length of time of the fixation and main sequences is equal. During the non-RSS session, the exact same procedure is used, except that each mask stimulus is replaced by a blank screen of equal duration (250 ms). EEG is recorded for the duration of each block. Testing was done in a dark shielded room. The viewing distance between participants and screen was 65 cm.

Task and organization varied across the 3 experiments. For the character-inversion study (experiment 1), both non-RSS and RSS sessions consisted of four blocks with 50 trials each block (100 trials for each type of character), 400 trials in total. The procedure for the face-inversion study (experiment 2), was similar to experiment 1 except that faces were used as target stimuli, instead of real Chinese characters. In the orientation experiments (1 and 2), participants were required to do a speeded orientation judgment task. They had to press “1” if the character or face was upright and press “2” when the character or face was inverted. For the character-lexicality study (experiment 3), participants judged “lexicality”—they were instructed to, as quickly and as accurately as possible, press key “1” with their index finger if they recognised the target character, or press key “2” with their middle finger if they did not. Real, pseudo and false targets were used, randomly intermixed. If a subject is making accurate judgments, real characters should be recognized, while pseudo and false characters should not. Both N170 and RP testing consist six blocks with 50 trials each block (100 trials for each type of character), 600 trials in total. In each paradigm, all stimuliwere mixed and randomised.

### EEG recording

Electroencephalogram were recorded from 30 scalp sites easy-Cap (Brian Product, Germany) with FCz as the default reference. In some subjects, 2 extra channels were included to record vertical electrooculographic (VEOG) activity and HEOG. The HEOG and VEOG electrodes were placed on the outer canthus of right eye and in the inferior areas of the left orbit, respectively. For subjects without ocular channels, eye blinks were recorded with the Fp1 and Fp2 electrodes. The ground electrode was placed along the midline, ahead of Fz, and impedances were systematically kept below 5 kΩ. Signals were digitized at a sampling rate of 500 Hz and band-pass filtered at 0.016~70 Hz. Participants were asked to minimize eye movement, head movement, and swallowing during the recording.

### Data analysis

EEG data were analysed in MATLAB 8.4.0.150421 (R2014b), using EEGLAB v13.4.4b [31], ERPLAB 5.0.0.0 [32], and customised scripts that made extensive use of these toolboxes. Data were first band-pass filtered at 0.1~30Hz, then separated into epochs -200 ms before target-stimulus onset to 600 ms after. Bad channels were detected with the automatic channel rejection function on EEGLAB and then replaced by interpolation from the remaining channels. ICA was implemented to correct eye blink artefacts. Baseline correction was done with-200~0ms. Epochs were rejected for abnormal trends if they had a slope larger than 75 μV/epoch and a regression R^2^ larger than 0.3; and rejected for amplitudes larger than 75 μV or smaller than -75 μV. ERP data were referenced to the average. ERP epochs were then averaged across trials within each condition for every subject. RP and N170 amplitudes were the main independent variable. RP/N170 peaks were detected using ERPLAB, as the most negative value within 150-250ms for non-RSS data, and 200 ms - 350ms for RSS data. These time windows were selected based on a careful visual inspection of individual subject waveforms: across subjects, the first negative deflection in the non-RSS conditions fell within 150-250ms post stimulus and the first negative deflection in the RSS conditions fell within 200-350ms post stimulus. Furthermore, the early and later windows we use are in broad agreement with the windows used in N170 and RP studies, respectively.

Statistical analyses were performed using R and the ezANOVA function [33–34]. In any case where the number of within-subject levels exceeded 2, Greenhouse-Geisser correction was used to adjust the degrees-of-freedom to correct for violations of the sphericity assumption, and epsilon values are reported [35]. In such cases, the adjusted p-values are reported. For all ANOVA, generalised eta-squared, ETA (junior epsilon with a hat), is reported as a measure of association strength for all significant effects [36–37]. N170 amplitude was examined in within-subject omnibus ANOVA: Paradigm x Stimulus.

Our evaluation of comparability of RP/N170 component between RSS and non-RSS paradigms included a comparison between the distributions of most-negative component across 10 posterior channels: O1, O2, Oz, Pz, P3, P4, P7, P8, TP9, and TP10. For these channels, we used time windows of 150–250 ms, and 200–350 ms, to find the most negative peak (across 10 channels) for N170 components in the non-RSS and RSS paradigms, respectively. These two time windows cover all the N1 peaks for grand-average non-RSS and RSS waveforms, respectively, at all 10 channels; while also excluding the P1 and P2 peaks. Additionally, these 2 time windows cover all the N1 peaks in each subject’s waveforms, respectively, for the 4 most critical channels: O1, O2, P7 and P8.

## Results

### Behavioral results

#### Experiment 1: Effect of orientation on characters

Mean accuracies and RT are reported in Tables 1 and 2, respectively.

**Table 1:** Mean accuracies for experiment 1, with standard deviation in parentheses.

		Stimulus	
		Upright	Inverted
Paradigm	Non-RSS	0.94 (0.04)	0.95 (0.04)
	RSS	0.92 (0.09)	0.91 (0.10)

**Table 2:** Mean reaction times for correct responses (ms) for experiment 1, with standard deviation in parentheses.

		Stimulus	
		Upright	Inverted
Paradigm	Non-RSS	550 (58)	562 (53)
	RSS	604 (56)	629 (49)

A repeated-measures 2 (Paradigm) x 2 (Stimulus) ANOVA of accuracy revealed a significant main effect of paradigm [F(1,23) = 5.4, p = 0.03, ETA = 0.047]. On average, accuracy was lower for the RSS paradigm. The mean difference of proportion correct (RSS minus non-RSS) was -0.031 [-0.05, -0.011].

A repeated-measures 2 (Paradigm) x 2 (Stimulus) ANOVA of reaction-time (RT) revealed significant main effects of paradigm [F(1,23) = 53, p = 2.1e-07, ETA = 0.25] and stimulus [F(1,23) = 29, p = 2e-05, ETA = 0.029], and an interaction [F(1,23) = 11, p = 0.0034, ETA = 0.0043]. Pair-wise t-tests comparing RSS and non-RSS paradigms were performed separately for upright and inverted stimuli. This demonstrated a significant non-RSS speed advantage for both upright and inverted stimuli. For upright stimuli: (t(23) = 5.8, p = 5.9e-06), with a 95-percent confidence interval of [0.035, 0.073] around a mean difference of 0.054 seconds. For inverted stimuli: (t(23) = 8.5, p = 1.5e-08), with a 95-percent confidence interval of [0.051, 0.084] around a mean difference of 0.067 seconds. All t-tests significant with Bonferroni correction (criterion p = 0.05/2 = 0.025).

#### Experiment 2: Effect of orientation on faces

Mean accuracies and RT are reported in Tables 3 and 4, respectively.

**Table 3:** Mean accuracies for experiment 2, with standard deviation in parentheses.

		Stimulus	
		Upright	Inverted
Paradigm	Non-RSS	0.94 (0.05)	0.94 (0.04)
	RSS	0.94 (0.07)	0.92 (0.07)

**Table 4:** Mean reaction times for correct responses (ms) for experiment 2, with standard deviation in parentheses.

		Stimulus	
		Upright	Inverted
Paradigm	Non-RSS	537 (62)	549 (58)
	RSS	584 (54)	600 (54)

A repeated-measures 2 (Paradigm) x 2 (Stimulus) ANOVA of accuracy revealed no significant effects.

A repeated-measures 2 (Paradigm) x 2 (Stimulus) ANOVA of reaction-time (RT) revealed significant main effects of paradigm [F(1,23) = 55, p = 1.5e-07, ETA = 0.16], and stimulus [F(1,23) = 16, p = 0.00052, ETA = 0.014], but no interaction. RSS was slower than non-RSS by a mean of 0.049 seconds [0.039, 0.059]. And inverted was slower than upright by a mean of 0.013 seconds [0.0074, 0.019].

#### Experiment 3: Effect of orthography on characters

Mean accuracies and RT are reported in Tables 5 and 6, respectively.

**Table 5:** Mean accuracies for experiment 3, with standard deviation in parentheses.

		Character type		
		Real	Pseudo	False
Paradigm	Non-RSS	0.91 (0.07)	0.84 (0.14)	0.99 (0.02)
	RSS	0.87 (0.10)	0.77 (0.23)	0.95 (0.06)

**Table 6:** Mean reaction times for correct responses (ms) for experiment 3, with standard deviation in parentheses.

		Character type		
		Real	Pseudo	False
Paradigm	Non-RSS	618 (50)	647 (67)	573 (54)
	RSS	664 (50)	696 (59)	641 (53)

A repeated-measures 2 (Paradigm) x 2 (Stimulus) ANOVA of accuracy revealed significant main effects of paradigm [F(1,23) = 20, p = 0.00019, ETA = 0.038], and stimulus [F(2,46) = 13, epsilon = 0.57, p = 0.00075, ETA = 0.23], but no interaction. The mean difference of proportion correct (RSS minus non-RSS) was -0.048 [-0.067, -0.028]. Two post-hoc paired t-tests were done to compare stimuli. Difference of proportion correct (pseudo-real) was significant: (t(47) = -2.8, p = 0.0073), with a 95-percent confidence interval of [-0.15, -0.024] around a mean difference of -0.086. Additionally, non-word minus pseudo proportion correct was also significant: (t(47) = 6.7, p = 2e-08), with a 95-percent confidence interval of [0.11, 0.21] around a mean difference of 0.16. All t-tests significant with Bonferroni correction (criterion p = 0.05/2 = 0.025).

A repeated-measures 2 (Paradigm) x 2 (Stimulus) ANOVA of RT revealed significant main effects of paradigm [F(1,23) = 56, p = 1.2e-07, ETA = 0.2], and stimulus [F(2,46) = 36, epsilon = 0.61, p = 5.2e-07, ETA = 0.19], and an interaction [F(2,46) = 7.8, epsilon = 0.8, p = 0.0029, ETA = 0.008]. Three post-hoc paired t-tests examined (RSS minus non-RSS) RT (seconds) separately for each stimulus type, and each was significant. Positive values of RT difference indicate that reactions were slower for the RSS paradigm compared to the non-RSS paradigm. Real words: (t(23) = 7, p = 3.5e-07), with a 95-percent confidence interval of [0.032, 0.059] around a mean difference of 0.046. Pseudo words: (t(23) = 5, p = 5.2e-05), with a 95-percent confidence interval of [0.029, 0.07] around a mean difference of 0.049. And non-words: The effect was significant (t(23) = 9.3, p = 2.8e-09), with a 95-percent confidence interval of [0.053, 0.083] around a mean difference of 0.068. All t-tests significant with Bonferroni correction (criterion p = 0.05/3 = 0.0167).

The results showed that responses in the non-RSS paradigm are significantly more accurate and rapid than in the RSS paradigm; except for accuracies measured in Experiment 2 (faces). Participants responded most accurately and rapidly for the false characters and least accurately and rapidly for the pseudo characters. Overall, our behavioral results demonstrate the effectiveness of our masks on perception, and the effectiveness of our stimulus manipulation on lexicality.

### ERP results

#### Comparability of RSS and non-RSS components

**Fig 2:**
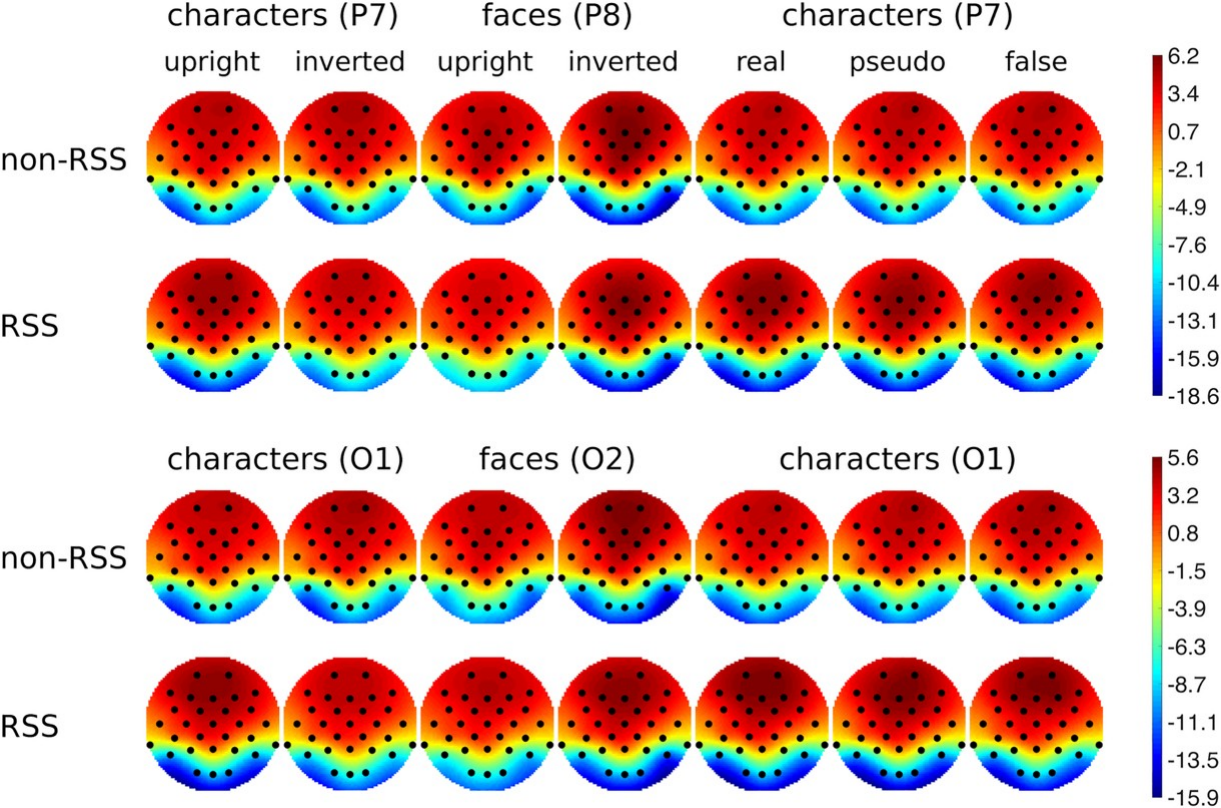
Grand average topographies at RP/N170 latency for the non-RSS paradigm (first and third rows), and at RP/N170 latency for RSS paradigm (second and fourth rows). First 2 columns show topographies at either P7 or P8 peaks (images scaled together); and last 2 columns at either O1 or O2 peaks (images scaled together). Conditions and experiments are arranged along columns: first 2 columns for the first experiment (character inversion), second 2 columns for the second experiment (face inversion), and final 3 columns for the third experiment (character orthography). Topographies were obtained for each subject, according to their specific peak latency, prior to averaging. Comparing across rows for each column reveals striking similarities between non-RSS- and RSS-derived topographical distributions.

In Fig 2, we show grand average topographies at RP/N170 latency for the non-RSS paradigm (first and third rows), and for the RSS paradigm (second and fourth rows). As one can see, the topographies are very similar, which is very encouraging. However, while this examination of grand-average topographies is typical in both N170 and RP literatures, some researchers have cautioned against making inferences from across-subject averages [38–39]. Therefore, the next 3 analyses of RP/N170 comparability avoid averaging across subjects.

**Fig 3:**
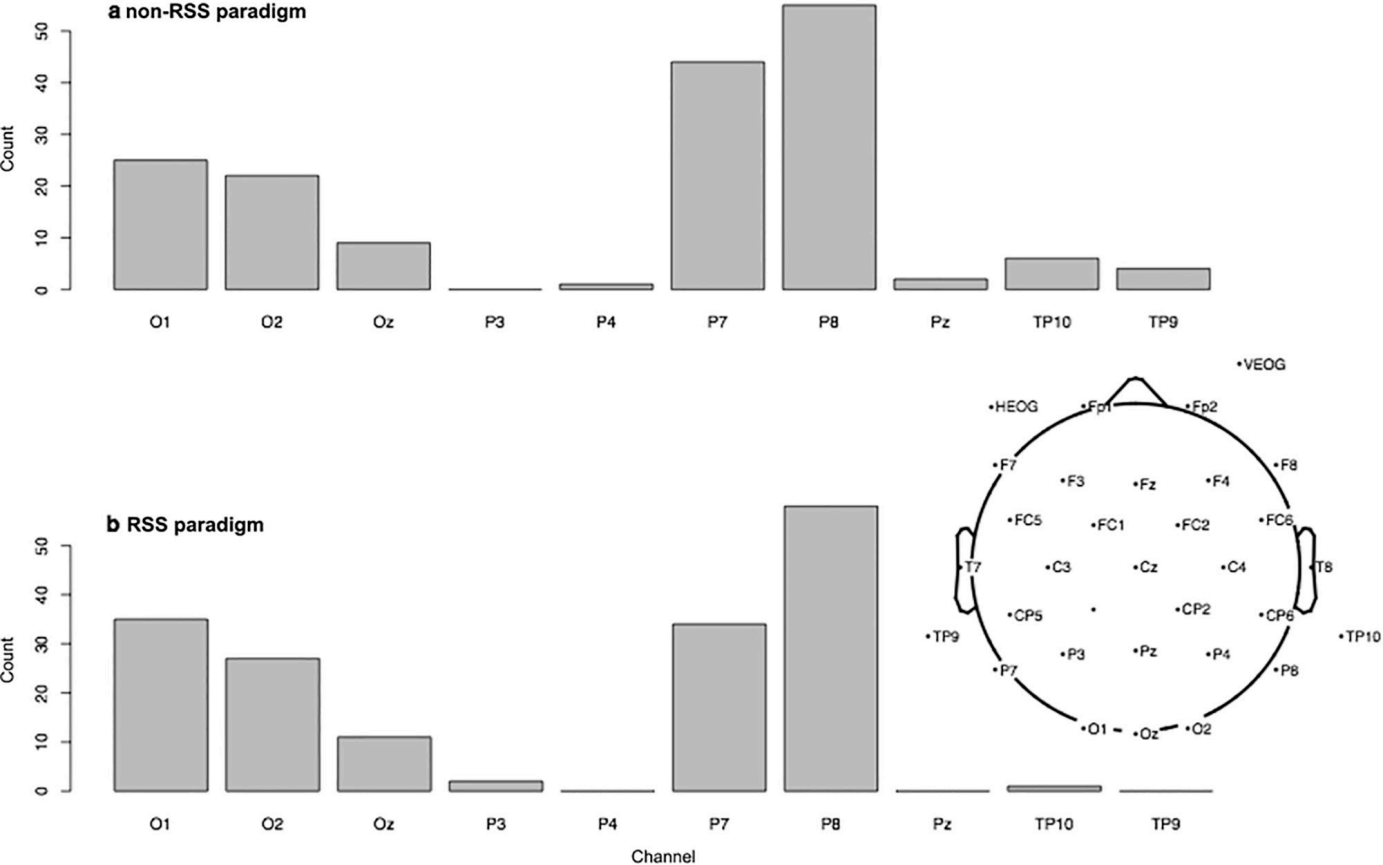
Histograms of posterior channels giving rise to the most negative non-RSS (**a**) or RSS peak (**b**), across all subjects, conditions and experiments. The distributions obtained from non-RSS (**a**) and RSS (**b**) paradigms are almost indistinguishable, except for a slightly lower relative frequency of P7 in the RSS paradigm. Time windows used for peak selection were 150–250 ms and 200–350 ms for non-RSS and RSS paradigms, respectively. Justification is provided in the Methods.

In the RP/RSS literature, a common way of identifying channels that best capture the RP component is to select channels (sometimes among a posterior subset) that give the most negative first deflection after stimulus onset [6–8, 10, 12–13, 23–24]. Even if other channels are included in the main ANOVA analyses, the most negative channel pair (left and right hemisphere) is usually isolated for post-hoc analyses. A similar channel-selection criterion is sometimes used in the N170 literature [40–41]. Therefore, another way to determine if N170 components are comparable between RSS and non-RSS paradigms, is to determine if these paradigms produce maximally-negative deflections in the same range of channels. Fig 3 shows histograms of posterior channels giving rise to the most negative non-RSS (a) or RSS peak (b), across all subjects, conditions and experiments. As one can see, the distributions look very similar for both paradigms. In particular, the most frequently negative channels are P7, P8, O1 and O2. It is important to note that the counts reported in Fig 3 are tallied across a combination of subjects, conditions and experiments. Therefore, the maximal attainable count is 168, which is 24 subjects multiplied by 7 types of stimulus: 2, 2 and 3 stimuli in experiments 1, 2 and 3, respectively.

**Fig 4:**
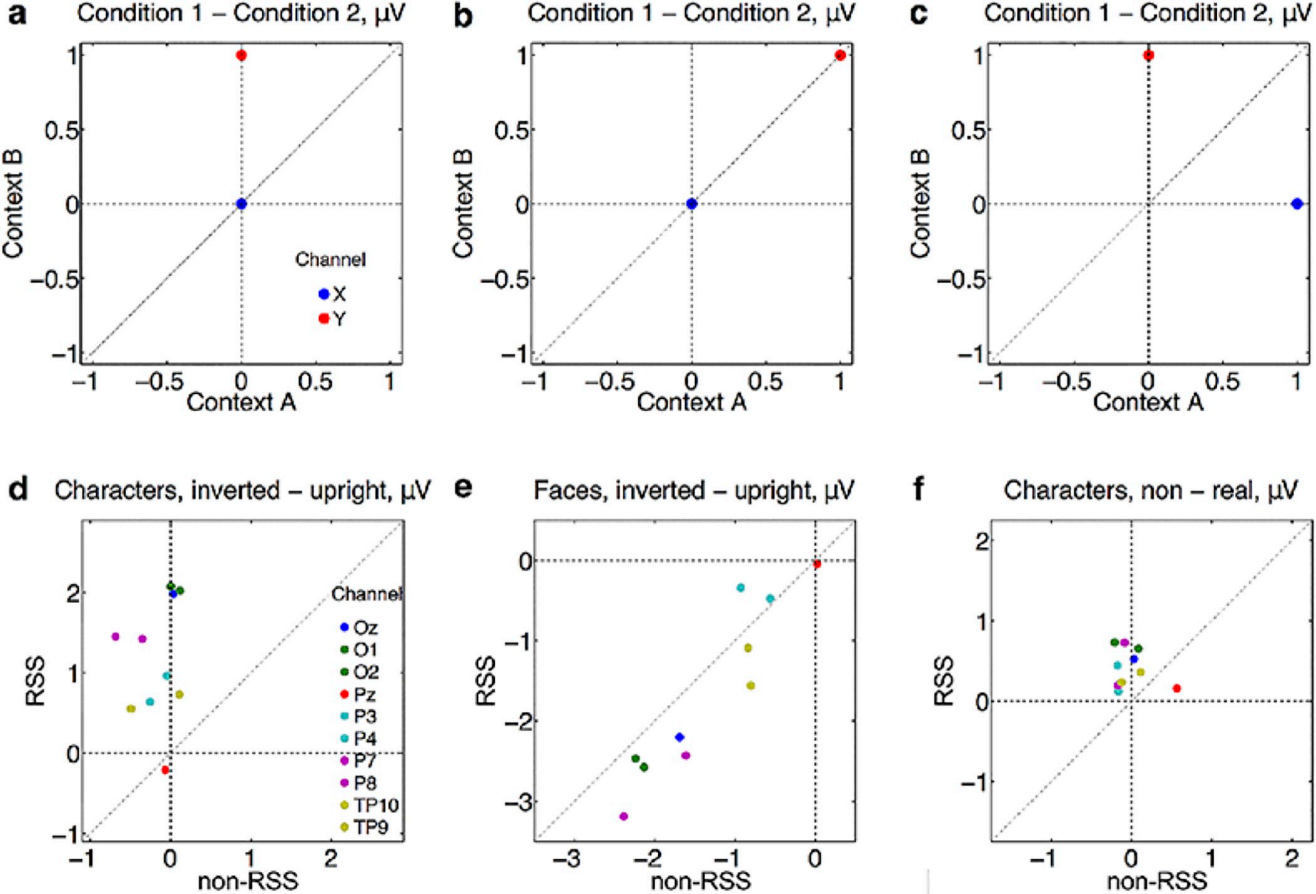
Scatterplots showing relation between stimulus selectivity for non-RSS and RSS paradigms, across 10 posterior channels; plots d, e, and f, for the 3 experiments, respectively. For each channel, in each plot, stimulus selectivities are averaged across subject. To interpret plots **d**, **e**, and **f**, we provide 3 hypothetical plots in **a**, **b**, and **c**, which show different ways in which the topographical distribution of stimulus-selectivity might change with paradigm. Plot a demonstrates orthogonal selectivity between paradigms, while b shows positively correlated selectivity. By orthogonal selectivity, we mean that there is selectivity in one situation (condition B in this case) and no selectivity in another situation (condition A in this case). These two scenarios suggest that a focus on channel Y is appropriate. However, the scenario depicted in plot c shows a negative correlation in selectivity, which suggests that selectivity exists in both paradigms, but in different channels; different channels should be examined for different paradigms. As shown by the real data plotted in d, e, and f, experiment 1 is consistent with orthogonal selectivity; experiment 2 with positively correlated selectivity; and experiment 3 with either orthogonal selectivity, or no selectivity (a scenario not depicted).

Perhaps more important than raw topography and component magnitude is stimulus selectivity. There is a possibility that non-RSS and RSS paradigms have selectivities that reside in different channel regions. However, we can see if this channel-switching actually occurs by examining the scatterplots shown in Fig 4, which displays both predictions (a, b, c) and our results (d, e, f). None of our three experiments are consistent with channel-switching of stimulus selectivity.

**Fig 5:**
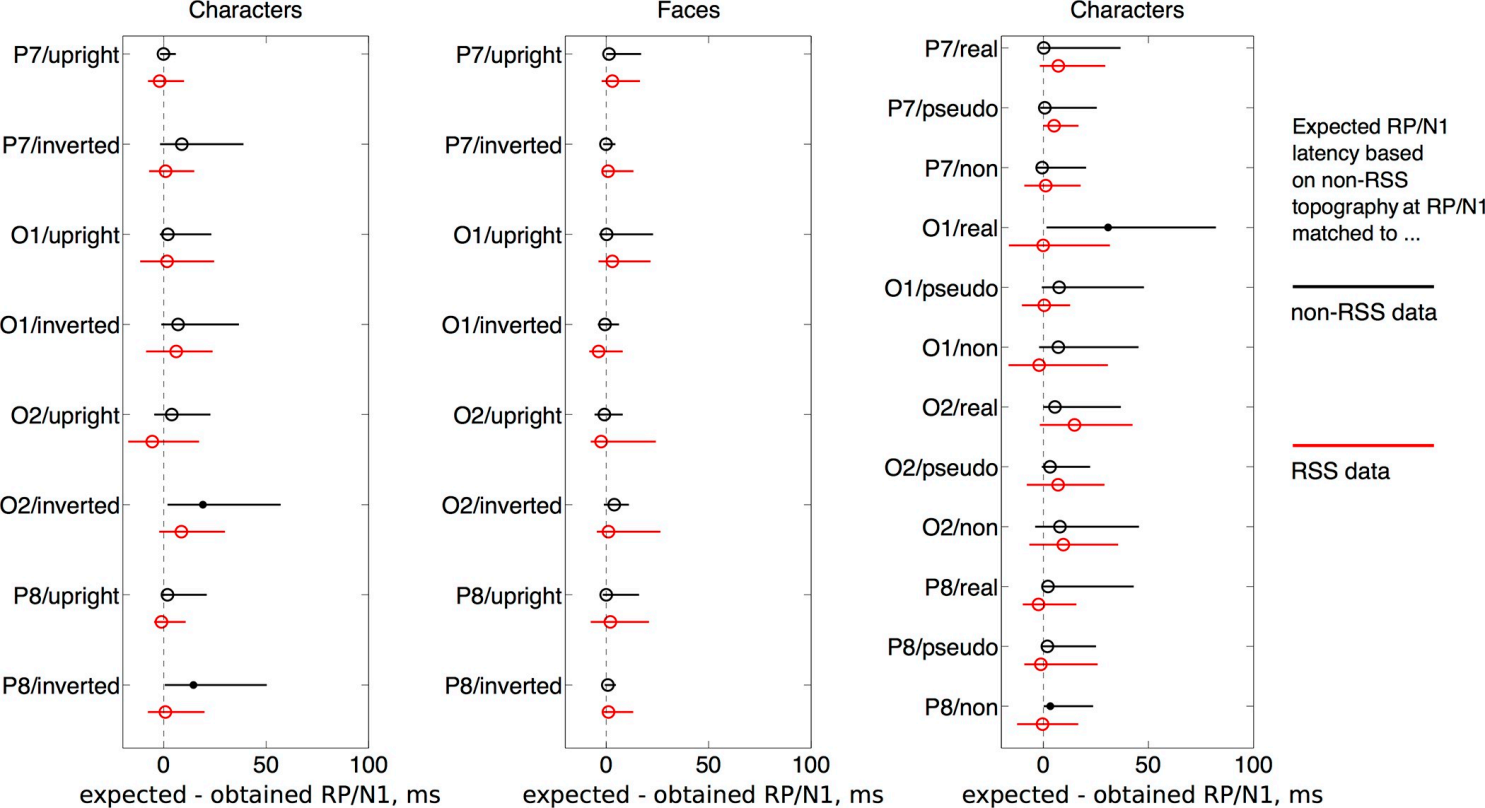
Ninety-five percent confidence intervals of 20-percent trimmed mean in difference between expected and obtained peak latencies, for non-RSS (black) or RSS (red) data. Expected peak latencies were those best matched to the topography of the RP/N170 peak (without noise masking) in the non-RSS data; based on maximum inner-product across latencies between 0 and 400 ms. Confidence intervals for non-RSS data (black) show how unique the topography of RP/N170 without noise masking is across time; while confidence intervals for RSS data (red) show how well the timing of the RP/N170 with noise masking is predicted by its topographical match to the N170 without noise masking. Black and red confidence intervals from the same condition/channel are always shown next to each other because we cannot expect red confidence intervals to be narrow if the corresponding black confidence intervals are wide. From left to right, the 3 plots show confidence intervals for experiments 1 to 3, respectively. Within each plot, pairs of confidence intervals (red and black) are given for different channel and stimulus combinations. Confidence intervals were obtained using a bootstrap procedure. Point estimates of the trimmed mean in latency difference are provided by circles; a filled circle for differences significantly greater than 0 (if the corresponding confidence intervals does not overlap with 0). In almost all cases, confidence intervals for RSS data are both near zero and narrow, relative to confidence intervals for non-RSS data.

Can the topography of the RP/N170, at the non-RSS latency, predict the topography at the RSS latency? For each subject, in each condition, of each experiment, we obtain the topography of the RP/N170 at non-RSS latency. Then we measure the inner-product between this non-RSS topography and every topography along that subject’s RSS time course. If non-RSS and RSS components are similar, then the maximal inner-product should occur at or around the RSS latency itself. Therefore, we expect differences between the expected RSS latency (based on inner-products with non-RSS topography) and the true RSS latency (based on the timing of the first negative deflection in RSS data) to be close to zero. Large temporal offsets may suggest that RSS and non-RSS are not comparable at the same channel. But another explanation is that the non-RSS topography is not unique within its own time course. Therefore, a baseline measurement of temporal offset should be considered: the difference between expected non-RSS latency and true non-RSS latency. Offsets for predicted RSS latency (between-paradigm inner-products) can only be as small as offsets for predicted non-RSS latency (within-paradigm inner-products). For all comparisons, we obtain ninety-five percent confidence intervals for the 20-percent trimmed mean of latency offsets, using a percentile bootstrap; a procedure with many advantages (Section 4.4.1 in Wilcox, 2011) [42]. Results are shown in Fig 5. As can be seen, each between-paradigm interval is similar in width, or less, to that obtained for its corresponding within-paradigm interval. And on an absolute scale, the intervals are certainly smaller than the intervals between successive peaks like the N1 and P2 (as seen in Fs 6 to 8, of the grand average waveforms).

### Experiment 1: Effect of inversion on N1 response to characters

**Fig 6:**
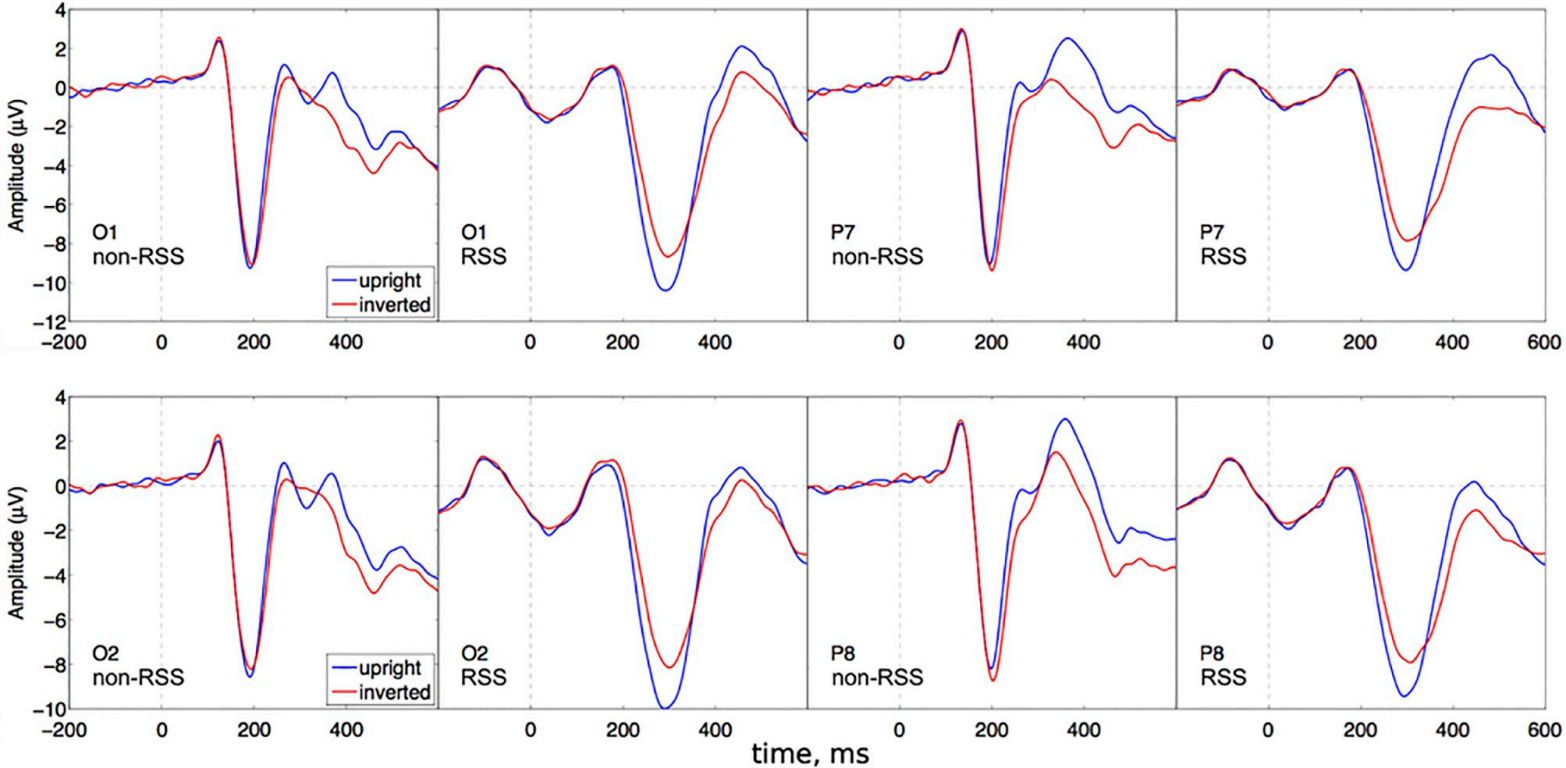
Grand average ERPs for experiment 1. For each of 4 posterior channels (O1, P7, O2, P8), ERP from non-RSS and RSS paradigms are shown in neighboring plots, and different stimulus conditions are shown on the same plot, using different colors. For each channel, the RSS paradigm appears to give greater stimulus selectivity relative to the non-RSS paradigm.

A repeated-measures 2 (Paradigm) x 2 (Stimulus) x 2 (Hemisphere) x 3 (Electrode) ANOVA revealed a significant main effect of electrode [F(2,46) = 42, epsilon = 0.77, p = 4.3e-09, ETA = 0.2], a significant main effect of stimulus [F(1,23) = 14, p = 0.001, ETA = 0.0039], a paradigm x stimulus interaction [F(1,23) = 32, p = 1e-05, ETA = 0.0074], a stimulus x electrode interaction [F(2,46) = 15, epsilon = 0.82, p = 4.8e-05, ETA = 0.0014], and a paradigm x stimulus x electrode interaction [F(2,46) = 17, epsilon = 0.97, p = 3.3e-06, ETA = 0.00084]. See Fig 6.

Following up on our 3-way interaction (paradigm x stimulus x electrode), we performed separate 2-way ANOVA (paradigm x stimulus) for each electrode group: O1/O2, TP9/TP10, and P7/P8. Those results led to a total of 6 post-hoc t-tests, so those tests were tested for significance against a criterion p (0.05 / 6 = 0.008), as per Bonferroni correction.

For O1/O2, there was both a main effect of stimulus [F(1,23) = 31, p = 1.2e-05, ETA = 0.0085], and a paradigm x stimulus interaction [F(1,23) = 34, p = 5.8e-06, ETA = 0.0076]. Pair-wise t-tests comparing upright and inverted characters were performed separately for non-RSS and RSS paradigms. This demonstrated a significant stimulus effect for RSS: (t(95) = 15, p = 5.1e-26), with a 95-percent confidence interval of [1.8, 2.3] around a mean difference of 2.1. However, no stimulus effect was found for the non-RSS paradigm: (t(95) = 0.49, p = 0.63), with a 95-percent confidence interval of [-0.19, 0.31] around a mean difference of 0.061.

The same result was found for TP9/TP10. A 2-way ANOVA (paradigm and stimulus) revealed a significant interaction [F(1,23) = 9.6, p = 0.0051, ETA = 0.0059]; and pairwise t-tests revealed significant stimulus effects for the RSS paradigm, but not for the non-RSS paradigm. The t-test for non-RSS paradigm gave (t(95) = -1.4, p = 0.16), with a 95-percent confidence interval of [-0.46, 0.076] around a mean difference of -0.19. While the t-test for RP gave (t(95) = 5.6, p = 2e-07), with a 95-percent confidence interval of [0.42, 0.87] around a mean difference of 0.64.

A slightly different result was obtained for P7/P8. The 2-way ANOVA (paradigm and stimulus) did reveal a significant interaction: [F(1,23) = 37, p = 3.3e-06, ETA = 0.0098]. However, t-tests comparing stimuli performed separately for non-RSS and RSS, both revealed a significant effect of stimulus. The t-test for non-RSS gave (t(95) = -3.5, p = 0.00079); and the t-test for RSS gave (t(95) = 10, p = 3.8e-17). Nonetheless, the magnitude of the stimulus effect was noticeably larger for RSS than for non-RSS: the 95-percent confidence interval and mean difference was [-0.82, -0.22] and -0.52 for non-RSS, while it was [1.2, 1.7] and 1.4 for RSS.

#### Experiment 2: Effect of orientation on N1 response to faces

**Fig 7:**
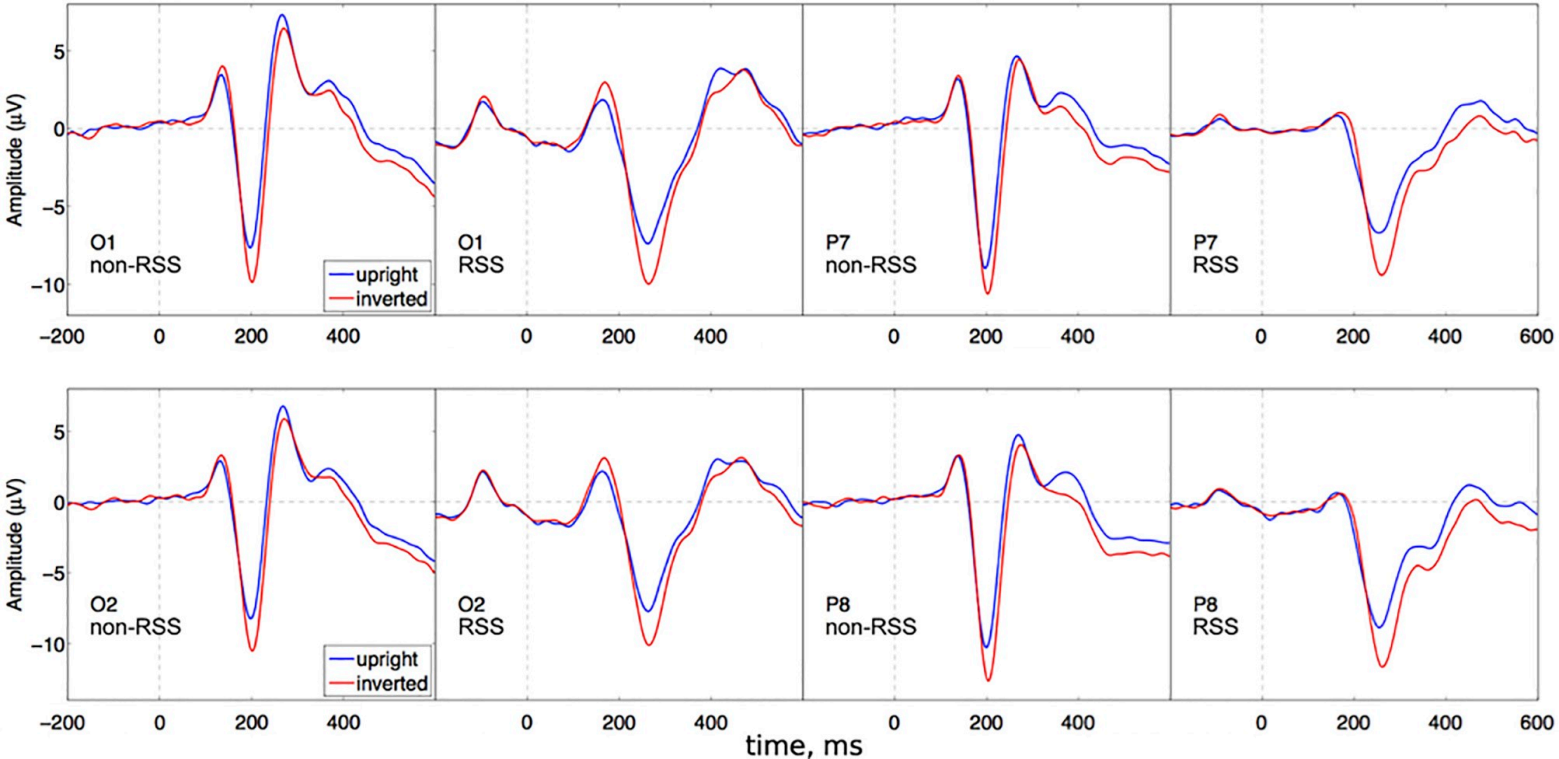
Grand average ERPs for experiment 2. Plots are arranged the same way as in Fig 6. Stimulus selectivity appears for each electrode, with no apparent modulation by paradigm.

A repeated-measures 2 (Paradigm) x 2 (Stimulus) x 2 (Hemisphere) x 3 (Electrode) ANOVA revealed a significant main effect of electrode [F(2,46) = 26, epsilon = 0.81, p = 3.8e-07, ETA = 0.18], stimulus [F(1,23) = 75, p = 1.1e-08, ETA = 0.043], hemisphere [F(1,23) = 13, p = 0.0014, ETA = 0.015], and a stimulus x electrode interaction [F(2,46) = 18, epsilon = 0.82, p = 1.2e-05, ETA = 0.0045]. See Fig 7.

Following up on our 2-way interaction (stimulus x electrode), we performed separate t-tests (stimulus) for each electrode: O1/O2, TP9/TP10, and P7/P8. While the magnitude of stimulus effects varied across electrode, a significant effect was obtained for each. For O1/O2: (t(191) = -17, p = 5.2e-41), with a 95-percent confidence interval of [-2.6, -2.1] around a mean difference of -2.4. For TP9/TP10: (t(191) = -11, p = 8e-21), with a 95-percent confidence interval of [-1.3, -0.87] around a mean difference of -1.1. And for P7/P8: (t(191) = -16, p = 2e-16), with a 95-percent confidence interval of [-2.7, -2.1] around a mean difference of -2.4. All t-tests were significant with Bonferroni correction (criterion p = 0.05/3 = 0.0167).

#### Experiment 3: Effect of orthography on N1 response to characters

**Fig 8:**
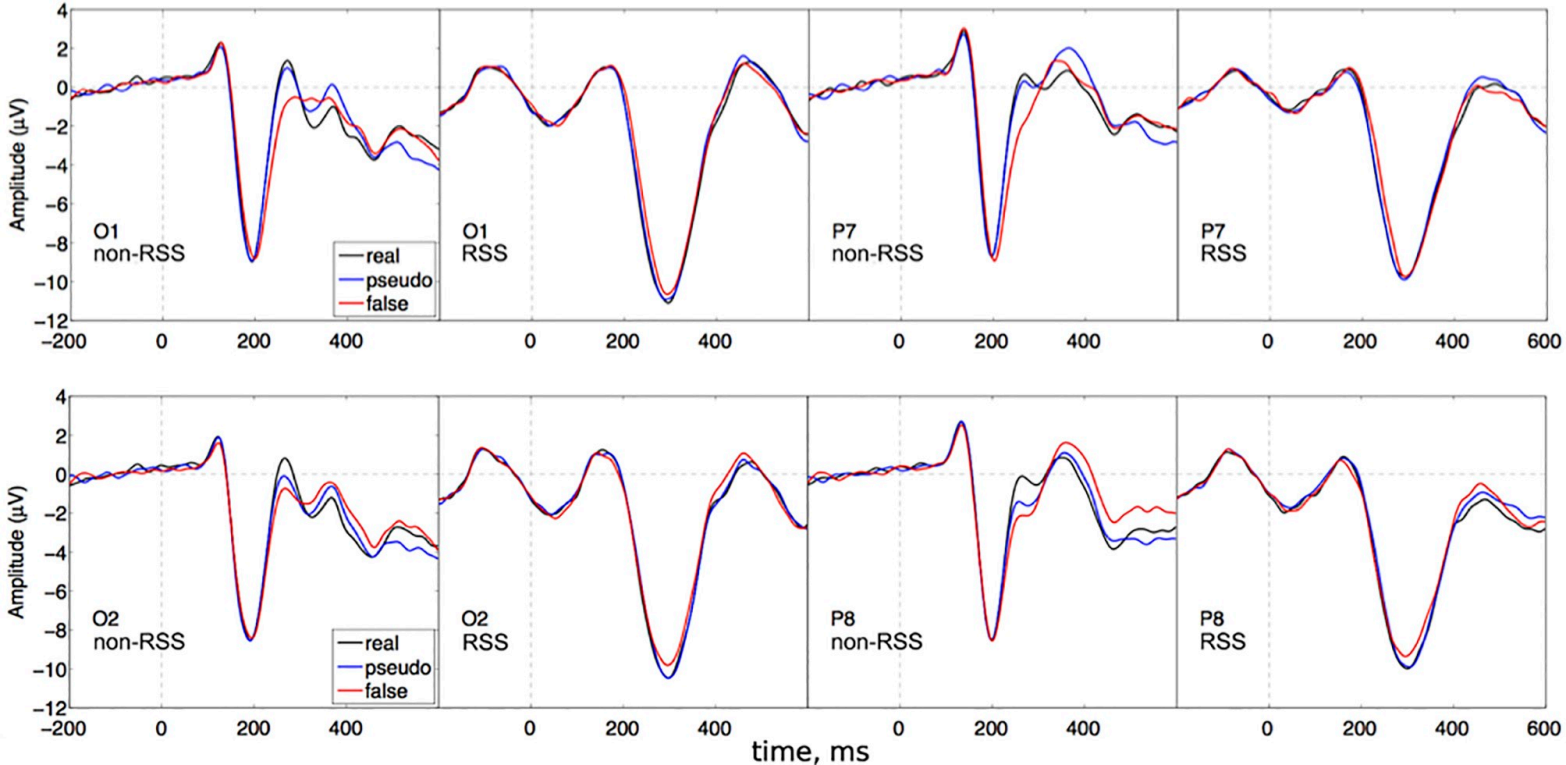
Grand average ERPs for experiment 3. Plots are arranged the same way as in Fig 6. Focusing on the difference between false characters and the other 2 types (possessing more semantic content), channels O2 and P8 suggest that the RSS paradigm gives rise to greater selectivity than in the non-RSS paradigm.

A repeated-measures 2 (Paradigm) x 2 (Stimulus) x 2 (Hemisphere) x 3 (Electrode) ANOVA revealed a significant main effect of stimulus [F(2,46) = 3.7, epsilon = 0.98, p = 0.033, ETA = 0.00048], electrode [F(2,46) = 40, epsilon = 0.75, p = 1.2e-08, ETA = 0.2], and a paradigm x stimulus interaction [F(2,46) = 3.6, epsilon = 0.96, p = 0.038, ETA = 0.00064]. See Fig 8.

Following up on our 2-way interaction (paradigm x stimulus), we performed separate 1-way ANOVA (stimuli: real, pseudo and false characters) for each paradigm (non-RSS and RSS).

While there was no significant effect of stimulus for the non-RSS paradigm, the RSS paradigm did reveal an effect: [F(2,46) = 8.3, epsilon = 0.92, p = 0.0012, ETA = 0.0055]. Two pairwise t-tests were performed to evaluate the stimulus differences obtained in the RSS paradigm: real versus pseudo characters, and pseudo versus false. Real characters did not produce significantly different amplitudes from pseudo characters: (t(287) = 0.46, p = 0.65), with a 95-percent confidence interval of [-0.076, 0.12] around a mean difference of 0.023. However, pseudo and non-characters were significantly different: (t(287) = 8, p = 2.5e-14), with a 95-percent confidence interval of [0.35, 0.57] around a mean difference of 0.46. We used Bonferroni correction (criterion p = 0.05/2 = 0.025).

#### Component latencies

Please see the S1 File. Our results replicate the previous finding that N170 latencies are delayed by face inversion [25–26]. Component latencies are not a key feature of the other studies we partially replicate in this study. So we refer the reader to the Supplemental Materials for our other findings regarding component latency.

## Discussion

This study presents the first systematic and fair comparison of both RP and N170 selectivity between RSS and non-RSS paradigms. We confirm prior assumptions about RSS by demonstrating that the RSS paradigm can enhance the stimulus selectivity of RP component amplitude in 2 different experiments that use Chinese characters as stimuli. Our enhanced ‘orthography effect’ in particular (experiment 3), may help to resolve contradictory results by showing noise-masking may be necessary to even obtain a stimulus selectivity. These positive results with RP responses to Chinese characters contrast with our face study, which shows that RSS does not enhance the N170 selectivity to face orientation. Unlike the familiar Chinese characters and radicals that we use in experiments 1 and 3, our Chinese faces were unfamiliar to our participants. Therefore, our results with unfamiliar faces is consistent with the notion that RSS enhances selectivity to aspects of the stimuli that are distinctly recognizable, and richer with semantic association. Finally, we include a complementary body of results, including some novel analyses, which addresses the important question of whether N1 components measured with and without RSS are comparable in the first place. Our results show that, at least for the stimuli we use (faces and Chinese characters), the answer is yes.

We took great care to ensure that the RP and N170 components we measured were similar enough between paradigms to be directly compared. First, we visualize grand-average topographies of RSS and non-RSS components, at their respective latencies, for each experiment and stimulus type. Second, we show separate histograms for RSS and non-RSS data, each showing the distribution of the most-negative first deflection across 10 posterior channels. Third, we use scatterplots to show how the stimulus-selectivity across channels changes between RSS and non-RSS paradigms. The second and third analyses help to determine if there is any ‘channel switching’ between RSS and non-RSS paradigms. We do not find any evidence of this. So, the final analysis is restricted to within-channel comparisons and focuses on the comparability of RSS and non-RSS timings. Indeed, RSS component latencies are well predicted by their topographical similarity to the non-RSS component. Importantly, these measures of topographical similarities (inner-products) are calculated separately for each subject and condition. Finally, as a complement to the preceding comparison between non-RSS and RSS components, our main result employs an ANOVA that includes both electrode and hemisphere as factors (6 electrodes across the posterior rim in total). RSS-enhancement suggests an interaction between stimulus (upright versus inverted, for example) and paradigm (RSS versus non-RSS). If masking also alters the topography of stimulus selectivity then that should emerge as an additional interaction with electrode or hemisphere (stimulus x paradigm x electrode, or hemisphere), and a specific pattern of post-hoc results suggesting that masking shifts selectivity to different electrodes, rather than simply amplifying selectivity at the same electrode(s). None of these analyses provided evidence that supports a difference of RP or N170 components between RSS and non-RSS paradigms.

The presence of object-related amplitude differences in the RP and N170 are the focal points of many electrophysiological studies. Much less discussed, or even reported, are the strength of those effects. Given that the main topic of this paper is an enhancement of ERP effects, we were careful to include effect sizes. We consider these results to be critical for the evaluation of all our effects. It is also useful to consider our RSS-based character inversion-effect in light of the prevailing notion for what is a strong ERP effect, and what is typically obtained for N170 inversion effects. Using RSS, we obtain an average inversion-effect of 2.1 [1.8, 2.3] for electrodes O1/O2 and 1.4 [1.2, 2.3] for P7/P8. In comparison, typical N170 studies often report significant stimulus selectivities that are based on a 1 microvolt difference (e.g. [18, 43]); while reports of the N170 inversion effect range from ~0.5 to 0.6 microvolts in two studies [44–45]; ~ -0.2 to 1.5 microvolt in one other study [46]. In some cases, it is clearly worth it to consider measuring a component using the RSS procedure.

Our character ‘orthography effect’ (pseudo versus false), while clearly enhanced by the presence of a forward mask, is a weaker effect compared to the inversion effect. Using RSS, this effect gives a mean difference of 0.46 microvolts with a confidence interval of [0.35, 0.57]. In this case, forward-masking may in fact be a requirement for obtaining an ‘orthography effect’. We feel that further examination of the character ‘orthography effect’ would greatly benefit from considering the inclusion of a forward mask; and perhaps manipulating the properties of that mask to better understand the specific nature of the stimulus selectivity that is being inferred.

We focused on the first negative deflection in this study (RP/N170) for 3 main reasons. First, our non-RSS conditions were replications of previous studies that focus on the first negative deflection. Second, almost all studies that employ RSS focus on the first negative deflection (RSS). And finally, other components like the P2 are difficult to isolate in many subjects; which is probably why there is such a strong focus on first negative deflections in the literature. However, the modest size of our ‘orthography effect’ also suggests that we might benefit from an examination of other components. Interestingly, Fig 8 suggests that there may be a strong effect of orthography on the second positive deflection, but only in the non-RSS condition. In the RSS condition, the second positive deflection is pushed beyond 400 ms. Therefore, it might be the case that the time-window around 200 ms is the critical factor in eliciting a reliable effect of orthography. Rather than examining individual peaks, the application of a linear model across matching time-points might be a better approach [38–39]. However, that type of analysis would constitute an entirely separate project, on account of the sheer volume of results and the lack of correspondence with previously published results. We are preparing these results for a separate publication.

Greater care needs to be taken when contrasting our N170 and RP results. As noted in the introduction, these components are often treated as different types of components. So, while both are the earliest negative deflection in response to recognizable objects, we adopt convention in labelling our character-evoked responses as RP and our face-evoked responses as N170. In our study however, another distinction may be of equal or greater importance. As our face stimuli were unfamiliar while our Chinese characters and radicals were familiar, recognizability was restricted to the category level in the former while it extended to exemplar level in the latter case. Therefore, the RSS enhancement of component selectivity may be restricted to stimuli that are familiar at an exemplar-specific level. However, while many studies show that RP (in response to words, characters and radicals) are modulated by exemplar-specific familiarity, there is little evidence that the N170 is modulated by face familiarity. We therefore, propose that RSS-enhancement of face selectivity might be found instead in the n250 component. Not only is this component modulated by face familiarity, it also peaks at around the same latency as the RP [40]. Of course, another possibility is that RSS-enhancement does not occur with N170 responses to faces because the evoked response without RSS is already so large. While that seems like the more parsimonious interpretation, additional study with familiar faces, and a study of the factors that may reduce N170 amplitude in non-RSS conditions, are required to draw any such conclusions.

A different approach to thinking about the difference between our N170 and RP results is to consider their neural generators. Different classes of stimuli like faces and written script may recruit different regions or networks of regions in the brain, which may vary in how RSS affects their sensitivity. Ideally, information about the neural generators of N170 and RP would derive from studies that use high spatial-resolution techniques combined with EEG. Unfortunately, such studies are not numerous. For example, Dien et al [9] is the only RP study to use fMRI and moreover, EEG and fMRI were measured separately. Nonetheless, we can draw the tentative conclusion that N170 and RP arise from different but nearby regions of the temporal cortex; the superior temporal sulcus [47] and inferior occipital gyrus [48] for N170, and the posterior inferior temporal gyrus for RP [9]. At the very least, those results can partially validate our focus on electrodes in the occipital-temporal regions of the scalp.

It is important to point out that the longer-term goal of this project is to investigate RSS as a tool for the enhancement and understanding of early component selectivity, in a very broad sense. We’ve demonstrated RSS-enhancement using 2 types of stimulus contrasts: upright versus inverted characters, and pseudo/real versus false characters. Those contrasts are important to vision and reading researchers for different reasons, but both may benefit from RSS-enhancement. This raises the question: What other types of early component selectivity can RSS enhance? And what, like the face-inversion effect, would remain unchanged by RSS? We feel that these empirical questions are of interest to the scores of other researchers who, like us, (1) may be uncertain about their ability to detect selectivity in their intrinsically noisy data; and (2) would like to understand the basis of this selectivity by identifying the factors that can modulate it. Of course, there are many ways to both obtain and analyze ERP data that might enhance the particular selectivity one is interested in. The RSS procedure is but one tool that can be used at the stage of stimulus presentation. Alternative forms of analysis (and experimental design) include the aforementioned linear modelling approach [38–39], component peaks (such as this study); logistic regression [49], reverse correlation [50], time-frequency analysis [51], subtraction of waveforms across hemispheres [52], and many more. Combining any of these analysis approaches with a stimulus-presentation approach such as RSS may lead to insights about the mechanism underlying stimulus selectivity. The value of an approach may very well depend on the particular stimulus or task space being studied and the underlying psychological processes involved, but that is ultimately an empirical question.

In all of our experiments, the sequence of stimulus-events, and their timing, were almost exactly matched between RSS and non-RSS trials. This strongly suggests that the enhanced inversion-effect and orthography-effect found for Chinese characters was due to the presence of a stream of masking stimuli. Furthermore, we used the same procedure to create the masking stimuli in both of our Chinese character experiments (experiments 1 and 3). However, our face experiment (2) did use a different procedure to generate masking stimuli. This does raise the possibility that our face masking stimuli could have been better designed to illicit RSS enhancement of N170 face selectivity. Pu et al. [24] show that RP amplitude can be directly related to the perceptual differences between mask and target. Perceptual differences between mask and target may have varied between our face and character experiments. However, there are other possibilities. Now that RSS-enhancement for Chinese characters has been demonstrated, perhaps it makes more sense to give serious consideration to the underlying mechanisms of this phenomenon. Two such mechanisms were briefly described in the introduction: the pre-empt explanation, and the visibility/cognitive-load trade-off. We will outline the relative merits of each.

Unlike the visibility/cognitive-load trade-off, the classic pre-empt explanation of RSS-enhancement takes into account the dynamic nature of RSS masking: masks and target-images are separated in time and this separation is easily perceived. Nonetheless, the effect of forward masking on the critical neural generators might be equivalent to the effect of simply degrading the target-image itself. Consistent with that possibility, RSS did reduce behavioral performance consistently across our experiments. To truly establish the importance of the dynamical aspect of the pre-empt explanation, one would need to model how ERP measured separately for mask and target images can be used to predict the response evoked by a sequence of mask and target. This level of explanation is currently lacking in the RSS/RP literature. On the other hand, an empirical verification of the visibility/cognitive-load trade-off would at least require confirmation of RSS-enhancement in conditions where the target-image is directly degraded and there is only a single evoked response isolated in time. This is easier to do than modeling ERP dynamics and it is something we are currently exploring. In the fMRI literature, there is some indirect support that masking-based enhancement can occur without RSS dynamics. Kay & Yeatman [53] measured BOLD responses in VWFA and FFA to both words and faces, as a function of both contrast and phase coherence. Lower phase coherence (less than 100-perent) mean greater amounts of image degradation. Kay & Yeatman [53] provide some evidence that intermediate levels of both contrast and phase-coherence resulted in greater BOLD responses to words compared to the maximum contrast and phase-coherence levels. Analogous to our own ERP results, this type of masking-based enhancement did not occur with face stimuli (see also [54]). It is encouraging that image degradation can enhance the BOLD response to words but not faces, just as RSS can enhance ERP to words but not faces in this study. Nonetheless, fMRI and EEG are very different. Moreover, the effect of image degradation on the BOLD response was not the primary focus of [53]. Therefore a replication of [53], and a separate study using EEG instead of fMRI would both be highly valuable contributions to understanding noise-enhancement.

In addition to the aforementioned projects, we believe that a fruitful way to study RSS-enhanced stimulus selectivity in the future would be to simply explore how masks may obscure the diagnostic features that are critical for the task and stimulus at hand. In this sense, the RSS procedure, together with a manipulation of mask characteristics, may lead to some insight into the information processing that underlies the generation of object-evoked potentials. With these results and insights in mind, we find great value in the continued investigation of RSS-enhancement. And, as the results of our orthography experiment suggest, greater care needs to be taken when comparing previous studies that differ in apparently trivial details like noise masking.

## Supporting information

S1 File This is the S1 File title. (PDF)
