# Supplementary material for: Rapid stream stimulation can enhance the stimulus selectivity of early evoked responses to written characters but not faces
Source: PLoS One. 2019 Mar 15;14(3):e0213637. doi: 10.1371/journal.pone.0213637 (PMC6420162; doi:10.1371/journal.pone.0213637)
Supplement: S1 File — (PDF) [file pone.0213637.s001.pdf]

Effect of inversion on latency of N1 response to characters

A repeated-measures 2 (Paradigm) x 2 (Stimulus) x 2 (Hemisphere) x 3 (Electrode) ANOVA revealed a significant main effect of electrode [F(2,46)=4.1, epsilon=0.81, p=0.032, ETA=0.011], paradigm [F(1,23)=1100, p=4.1e-21, ETA=0.88], and stimulus [F(1,23)=5.7, p=0.025, ETA=0.01]; a paradigm x electrode interaction [F(2,46)=10, epsilon=0.67, p=0.0015, ETA=0.029], and a paradigm x stimulus x electrode interaction [F(2,46)=4.7, epsilon=0.77, p=0.023, ETA=0.0055].

Following up on our 3-way interaction (paradigm x stimulus x electrode), we performed separate 2-way ANOVA (paradigm x stimulus) for each electrode group: O1/O2, TP9/TP10, and P7/P8. Those results led to a total of 2 post-hoc t-tests, so those tests were tested for significance against a criterion p (0.05 / 2 = 0.025), as per Bonferroni correction.

For O1/O2, there was a main effect of paradigm [F(1,23)=730, p=6.7e-19, ETA=0.91]. RSS minus non-RSS latencies (ms) had a 95-percent confidence interval of [99.2, 105.3] around a mean of 102.3.

For TP9/TP10, there was a main effect of paradigm [F(1,23)=670, p=1.8e-18, ETA=0.82], and a paradigm x hemisphere interaction [F(1,23)=4.7, p=0.041, ETA=0.016]. Pair-wise t-tests comparing RSS and non-RSS paradigms were performed separately for left and right hemispheres. This demonstrated a significant paradigm effect for both hemispheres. RSS minus non-RSS latency (ms) was significantly different from 0 in the left hemisphere: (t(95)=25, p=3.1e-44), with a 95-percent confidence interval of [77, 90] around a mean latency difference of 83. RSS latencies were also significantly delayed in the right hemisphere: (t(95)=44, p=1.1e-64), with a 95-percent confidence interval of latency difference (ms) of [90, 98] around a mean difference of 94.

For P7/P8, there were main effects of paradigm [F(1,23)=1000, p=1.2e-20, ETA=0.91], and stimulus [F(1,23)=13, p=0.0015, ETA=0.033]. RSS minus non-RSS latencies (ms) had a 95-percent confidence interval of [98, 100] around a mean of 100. Inverted- minus upright-character latencies (ms) had a 95-percent confidence interval of [3.6, 7.8] around a mean of 5.7.

Effect of orientation on latency of N1 response to faces

A repeated-measures 2 (Paradigm) x 2 (Stimulus) x 2 (Hemisphere) x 3 (Electrode) ANOVA revealed a significant main effect of paradigm [F(1,23)=580, p=7.7e-18, ETA=0.82], and stimulus [F(1,23)=15, p=0.00078, ETA=0.021]. RSS minus non-RSS latencies had a 95-percent confidence interval of [58, 61] around a mean of 60. Inverted- minus upright-character latencies (ms) had a 95-percent confidence interval of [3, 5] around a mean of 4.

Effect of orthography on latency of N1 response to characters

A repeated-measures 2 (Paradigm) x 2 (Stimulus) x 2 (Hemisphere) x 3 (Electrode) ANOVA revealed a significant main effect of stimulus [F(2,46)=3.6, epsilon=0.79, p=0.047, ETA=0.0042], paradigm [F(1,23)=1000, p=1.4e-20, ETA=0.88], and a significant paradigm x hemisphere interaction [F(1,23)=7.1, p=0.014, ETA=0.011].

Following up on our main effect of stimulus, we conducted a t-test for each of the 3 pair-wise combinations of real, pseudo and false characters. Following up on our 2-way interaction (paradigm x hemisphere), we performed separate t-tests comparing RSS to non-RSS for each hemisphere. Taken

together, this constitutes a total of 5 post-hoc t-tests, so those tests were tested for significance against a criterion p (0.05 / 5 = 0.01), as per Bonferroni correction.

Of the 3 pairwise comparisons among stimulus types, only the N1 responses to false characters were significantly delayed compared to real characters: (t(575)=4.1, p=4.1e-05), with a 95-percent confidence interval of latency difference (ms) of [1.5, 4.1] around a mean difference of 2.8.

In the left hemisphere, RSS latencies were significantly longer than non-RSS latencies: (t(431)=74, p=4.7e-248), with a 95-percent confidence interval of latency difference (ms) of [94, 89] around a mean difference of 91. In the right hemisphere, RSS latencies were significantly longer than non-RSS latencies: (t(431)=88, p=9.9e-277), with a 95-percent confidence interval of latency difference (ms) of [100, 97] around a mean difference of 99.
